# Phosphatase UBLCP1 controls proteasome assembly

**DOI:** 10.1098/rsob.170042

**Published:** 2017-05-24

**Authors:** Shuangwu Sun, Sisi Liu, Zhengmao Zhang, Wang Zeng, Chuang Sun, Tao Tao, Xia Lin, Xin-Hua Feng

**Affiliations:** 1 Life Sciences Institute and Innovation Center for Cell Signaling Network, Zhejiang University, Hangzhou, Zhejiang, People's Republic of China; 2 Michael E. DeBakey, Department of Surgery, Houston, TX, USA; 3 Department of Molecular and Cellular Biology, Baylor College of Medicine, Houston, TX, USA; 4 State Key Laboratory of Stress Cell Biology, School of Life Sciences, Xiamen University, Xiamen, Fujian, People's Republic of China

**Keywords:** UBLCP1, phosphorylation, proteasome, CTD phosphatase

## Abstract

Ubiquitin-like domain-containing C-terminal domain phosphatase 1 (UBLCP1), an FCP/SCP phosphatase family member, was identified as the first proteasome phosphatase. UBLCP1 binds to proteasome subunit Rpn1 and dephosphorylates the proteasome *in vitro*. However, it is still unclear which proteasome subunit(s) are the *bona fide* substrate(s) of UBLCP1 and the precise mechanism for proteasome regulation remains elusive. Here, we show that UBLCP1 selectively binds to the 19S regulatory particle (RP) through its interaction with Rpn1, but not the 20S core particle (CP) or the 26S proteasome holoenzyme. In the RP, UBLCP1 dephosphorylates the subunit Rpt1, impairs its ATPase activity, and consequently disrupts the 26S proteasome assembly, yet it has no effects on the RP assembly from precursor complexes. The Rpn1-binding and phosphatase activities of UBLCP1 are essential for its function on Rpt1 dephosphorylation and proteasome activity both *in vivo* and *in vitro*. Our study establishes the essential role of the UBLCP1/Rpn1/Rpt1 complex in regulating proteasome assembly.

## 1. Introduction

The ubiquitin proteasome system (UPS) plays a key role in maintaining cellular proteostasis [1]. Aberrant proteostasis may lead to cell dysfunction and diseases such as cancer and neurodegenerative disorders. A functional 26S proteasome comprises a barrel-shaped 20S core particle (CP) [1,2] and one or two 19S regulatory particles (RP) capping at the ends [2,3]. The CP is stacked by two outer α-rings and two inner β-rings. The RP is further divided into ‘lid' and ‘base', and the base consists of a hetero-hexameric AAA-ATPase ring (Rpt1–6) and non-ATPase subunits Rpn1, Rpn2 and Rpn13 [3–6]. Poly-ubiquitinated protein substrates are first recognized by RP through ubiquitin receptors or ubiquitin shuttle receptors in the RP [2,5,6]. Ubiquitin chains are further cleaved off by deubiquitinases USP14, Uch37 and Rpn11 on RP [7,8]. Besides, RP is also responsible for substrate unfolding and CP α-ring gate opening in an ATP-dependent manner [9–11]. In the end, the substrates committed to degradation will be translocated into the hydrolytic chamber of the CP for degradation.

The cellular proteasome is a complex with persisting dynamic assembly and disassembly [12]. Although the process of mammalian proteasome assembly is controversial, it is generally believed that RP and CP are assembled independently, and then associated with each other to form a functional proteasome holoenzyme. The CP assembly is initiated with α-ring formation and completed with a cylinder-shaped particle that contains proteases β1, β2 and β5 [12,13]. The process of the RP lid assembly is still poorly understood. However, it is well known that RP base is assembled from three precursor subcomplexes, which are heterodimeric Rpt pairs, Rpn1-Rpt1-Rpt2-S5b, p28/gankyrin-Rpt3-Rpt6-PAAF1 and p27-Rpt4-Rpt5 [2,12,14]. S5b, p28/gankyrin, PAAF1 and p27 are the four known RP assembly chaperones, which sequentially participate in RP assembly and detach from mature 26S proteasome. Loss of these chaperones leads to proteasome assembly defect. RP base, the subcomplex coupling substrate binding and substrate degradation, has an essential role in proteasome activity and function. Specifically, the AAA-ATPase ring on RP plays a key role in the process. Upon ATP binding, proteasome ATPase activates CP gate opening by inserting the C-terminal hydrophobic-tyrosine-X (HbYX) motif of Rpt2, Rpt3 and Rpt5 into the ‘pocket' formed by the CP α-ring [5,15] and significantly enhances the affinity of ubiquitinated substrate to the RP [16]. The lid and base are associated with each other to complete the RP assembly, which also requires the catalytic activity of ATPase subunits Rpt1–6 [17].

Protein function is largely regulated by posttranslational modifications (PTMs) [18–20]. Phosphorylation, the most prevalent PTM, of proteasome subunits has been reported in both CP and RP. There are several reports describing phospho-regulation of proteasome by kinases, such as cAMP-dependent protein kinase (PKA) and Ca^2+^-calmodulin-dependent protein kinase 2α (CaMKIIα) [19,21–29]. For example, PKA phosphorylates Rpt6 at Ser^120^ leading to enhanced proteasome assembly and activity [21,29]. UBLCP1 was identified as the first phosphatase associated with the proteasome [30]. UBLCP1 features the N-terminal ubiquitin-like (UBL) domain, which interacts with the proteasome [31], and the CTD phosphatase domain at its C-terminus. Subsequently, Guo and co-workers [32,33] reported that UBLCP1 directly interacts with the proteasome and dephosphorylates proteasome *in vitro*. In contrast with PKA's function on the proteasome, UBLCP1 impairs proteasome assembly and activity. However, it is still unclear which proteasome subunit(s) are the *bona fide* substrate(s) of UBLCP1 and the precise mechanism for proteasome regulation remains elusive.

During our extensive studies on protein phosphatases [34–36], we independently found that UBLCP1 physically interacts with proteasome subunit Rpn1. Our study agrees with the report by Guo and co-workers [32,33] that UBLCP1 binds to RP and disrupts the 26S proteasome assembly. We further show that UBLCP1 exerts these effects both *in vivo* and *in vitro*, and has no effects on RP assembly. Through its interaction with Rpn1, UBLCP1 dephosphorylates the RP subunit Rpt1 and impairs its ATPase activity, whereas the phosphatase-dead mutant of UBLCP1 with Asp-to-Ala substitution at Asp^143^ and Asp^145^ (DDAA) fails to exert these functions. Notably, Rpn1 is essential for the function of UBLCP1 on Rpt1 dephosphorylation and proteasome activity in the cell. Our study establishes the essential role of the UBLCP1/Rpn1/Rpt1 complex in regulating the proteasome assembly and activity.

## 2. Material and methods

### 2.1. Plasmids and antibodies

DNA fragments encoding proteasome subunits, chaperones, Rad23B and USP14 were amplified from corresponding cDNAs in the human ORFeome collection (v.5.1) and subcloned into pXF6F (3 × FLAG) vector (derived from pRK5, Genentech). HA-, MYC- and GST-tagged UBLCP1 were generated by PCR from UBLCP1 cDNA and subcloned into pXF4H vector (derived from pRK5, Genentech), pXF3HM vector (our laboratory) and pGEX-4T-1 (GE Healthcare), respectively. The deletions and point mutants of UBLCP1 were also generated by PCR. NLS-GFPu plasmid was kindly provided by Ron Kopito (Stanford University, CA, USA). The antibodies were purchased as follows: anti-α1–7 (#ST1049) and anti-phosphoserine (Phosphoserine Detection Kit, #525282) antibodies from EMD Millipore; anti-Rpn1 (#HPA045192), anti-FLAG (#F3165) and anti-γ-tubulin (#T5326) antibodies from Sigma; anti-UBLCP1 (#ab176340) and anti-histone H3 (#ab1791) antibodies from Abcam; anti-MYC (#sc-40), anti-GFP (#sc-8334) and anti-Cyclin D1 (sc-753) antibodies from Santa Cruz; anti-HA (#3724) and anti-p21^Cip1^ (#2947) antibodies from Cell Signaling Technology; anti-Rpn2 (#PW 9270), anti-Rpn7 (#PW8225), anti-Rpt1 (#PW8825), anti-Rpt3 (#PW8175), anti-Rpt4 (#PW8220), anti-Rpt5 (#PW8770) and anti-Rpt6 (#PW8320) antibodies from ENZO Life Sciences; peroxidase-conjugated goat anti-rabbit and rabbit anti-mouse IgG (#315-035-048) from Jackson ImmunoResearch.

### 2.2. Cell lines, transfection and stable cell line generation

Human HEK293T and HeLa Tet-Off cells were grown as described previously [37]. HEK293T cells stably expressing HTBH-tagged (Histidine-TEV cleavage sequence-Biotin-Histidine) Rpn11 were reported previously [38]. HeLa cells were transfected with X-tremeGENE (Roche). HEK293T cells were transfected with PEI (Polyscience).

### 2.3. RNA interference and *hSpCas9*-mediated knockout

Small interference RNAs targeting human UBLCP1 and Rpn1 were transfected into cells for 24 h using Lipofectamine RNAiMAX Reagent (Invitrogen). The oligo siUBLCP1 #1 (nt 420–438 of coding sequence (CDS), GGTGCTAGATGTTGATTAT), siRpn1 #1 (nt 171–189 of CDS, GATGCTCGTGGAACGACTA), siRpn1 #2 (nt 1594–1612 of CDS, GGAGATGTAACTTCCACTA) and siRpn1 #3 (nt 2235–2253 of CDS, GCGCCAGTTAGCTCAATAT) were provided by RiboBio Co. Corresponding DNA sequences for generating shUBLCP1 (target sequence: nt 820–840 of CDS, GCGCACCTAAATCGTGATAAA) were cloned into pSRG vector for stable UBLCP1 knockdown. Stable HEK293T and HeLa cell lines expressing shUBLCP1 or carrying pSRG vector were generated and selected with 1 μg µl^−1^ of puromycin (Sigma). The target sequence of UBLCP1 gRNA (nt 652–671 of genome, ACAGTACATACTCCAAGGAG) was used to clone into pX330 vector for *hSpCas9*-mediated knockout in HEK293T cells.

### 2.4. Immunoprecipitation, western blotting and mass spectrometry analysis

Twenty-four hours after transfection, cells were harvested by FLAG lysis buffer (300 mM NaCl, 25 mM Tris–HCl (pH 7.5), 2 mM EDTA and 1% Triton X-100, protease inhibitor cocktail (aprotinin, leupeptin and phenylmethylsulfonyl fluoride)) and incubated with Protein A Sepharose CL-4B (GE Healthcare) and appropriate antibodies for 4 h. After extensive washes, immunoprecipitated proteins were boiled in SDS sample loading buffer, separated by SDS-PAGE or Phos-tag gel, transferred onto PVDF membranes (Millipore), and detected by western blotting using appropriate antibodies. Lysates from HEK293T cells stably overexpressing FLAG-UBLCP1 were subjected to immunoprecipitation using anti-UBLCP1 antibody or control rabbit IgG. Immunoprecipitation products were subjected to mass spectrometry analysis.

### 2.5. *In vitro* translation and GST pull-down assay

*In vitro* translation was performed with the TNT Quick Coupled Transcription/Translation System (Promega). GST-fused proteins were expressed in *Escherichia coli* BL21 (DE3) strain and purified according to manufacturer's instructions. GST pull-down experiments were carried out as previously described [37].

### 2.6. *p*-Nitrophenyl phosphate assay

Recombinant GST-UBLCP1, GST-UBLCP1-DDAA or GST protein was added to a reaction mixture containing 50 mM Tris–acetate (pH 5.0), 10 mM MgCl_2_, 20 mM *p*-nitrophenyl phosphate (*p-*NPP, AMRESCO) and then incubated at 37°C for 1 h. The reaction was quenched by adding 2 M sodium carbonate. Production of *p*-nitrophenyl was monitored by measuring the absorbance at 405 nm.

### 2.7. Nuclear fractionation

HEK293T cells were harvested by PBS. After gentle PBS-wash, cell pellets were resuspended in buffer A (10 mM HEPES (pH 7.5), 10 mM KCl, 1.5 mM MgCl_2_, 1 mM DTT, 1 mM ATP and 20 mM NaF) by pipetting and incubated on ice for 5 min. The buffer was then changed with buffer A containing 0.003% digitonin (Sigma). After a short centrifugation, the cell pellets were quickly washed twice with 3 ml buffer A containing 0.003% digitonin. Finally, the nuclear pellets were lysed with FLAG lysis buffer.

### 2.8. GST-UBL^hRad23B^ affinity purification of 26S proteasome

Affinity purification of mammalian 26S proteasomes was carried out according to a previous study [39]. Recombinant GST-UBL^hRad23B^ (UBL domain of hRad23B) protein and His_3_-UIM protein were expressed in *E. coli* BL21 (DE3) strain and purified according to the manufacturer's instructions. HEK293T cells were lysed with affinity purification buffer (APB; 25 mM HEPES–KOH (pH 7.5), 10% glycerol, 5 mM MgCl_2_, 1 mM ATP and 1 mM DTT) containing 0.5% Nonidet *P*-40. The HEK293T cell lysates were incubated with purified GST-UBL^hRad23B^ protein on glutathione agarose beads (Pierce) for 4 h. After extensive washes with APB buffer, the affinity-purified proteasomes were eluted with His_3_-UIM or boiled with SDS sample loading buffer.

### 2.9. Purification of free RP/CP and proteasome reconstitution *in vitro*

Free RP purification was done essentially as previously described [40]. Briefly, streptavidin–agarose beads (Invitrogen) were used to pull-down proteasome from HEK293T cells stably expressing HTBH-tagged Rpn11. The beads were incubated with high salt buffer (5 mM HEPES–KOH (pH 7.5), 10% glycerol, 5 mM MgCl_2_, 500 mM NaCl) at 4°C for 1 h to disrupt the RP–CP association. After extensive wash with APB buffer, the RP was removed from the beads by adding TEV protease. Free CP was purified from HEK293T cells transfected with FLAG-tagged β4. FLAG beads (Sigma) were used to purify FLAG-tagged CP. The washing condition was the same as RP purification. Finally, FLAG-tagged CP was eluted with a FLAG peptide (Sigma). The purity of RP and CP was evaluated with SDS-PAGE following silver staining (Beyotime). The proteasome was constituted by mixing RP and CP at a ratio (molar) of 3 : 1 in reconstitution buffer (50 mM Tris–HCl (pH 7.5), 5 mM MgCl_2,_ 2 mM ATP) at 30°C for 30 min.

### 2.10. Proteasome activity measurement and in-gel peptidase assay

Proteasome activity measurement was done as previously reported [41]. Briefly, purified proteasomes or cell lysates were incubated with 100 µM Suc-LLVY-AMC (ENZO Life Sciences) in a proteasome activity buffer (50 mM Tris–HCl (pH 7.5), 40 mM KCl, 5 mM MgCl_2_, 1 mM DTT, 2 mM ATP). The free AMC fluorescence can be quantified with an excitation at 380 nm and emission at 460 nm. For in-gel peptidase assay, the purified nuclear proteasome was resolved on 3.5% native PAGE. The native PAGE was then incubated with 100 µM Suc-LLVY-AMC in the proteasome activity buffer. AMC release was visualized under UV light.

### 2.11. ATPase activity assay

Cells expressing FLAG-tagged proteasome ATPase subunits were subjected to immunoprecipitation using anti-FLAG antibody. Immunoprecipitation products were incubated with ATP at 37°C for 30 min. ADP production was measured by ADP-Glo assay (Promega). Luminescence was read using a luminometer.

## 3. Results

### 3.1. UBLCP1 selectively binds to regulatory particle

**Figure 1.**
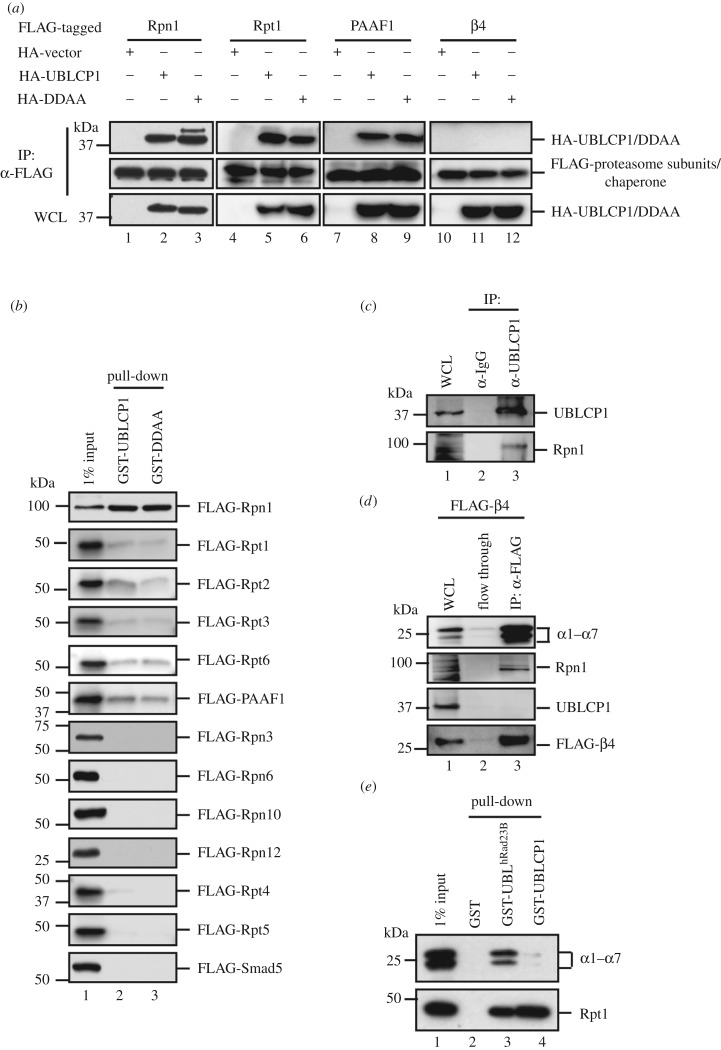
UBLCP1 selectively binds to RP. (*a*) Co-immunoprecipitation (co-IP) of HA-UBLCP1 with selected FLAG-tagged proteasome subunits and PAAF1 in HEK293T cells. IP, immunoprecipitation; IB, western blotting; WCL, whole-cell lysates; DDAA, a UBLCP1 mutant with Asp-to-Ala substitution at Asp^143^ and Asp^145^. (*b*) Interaction of purified recombinant GST-UBLCP1 or GST-DDAA with *in vitro* translated FLAG-tagged RP subunits from rabbit reticulocyte lysate. GST-UBLCP1 bound proteasome subunits were detected by western blotting with anti-FLAG antibody. (*c*) Endogenous UBLCP1–Rpn1 interaction. HEK293T cells were collected, and cell lysates were subjected to IP using anti-UBLCP1 antibody or control rabbit IgG. IP products were probed with indicated antibodies. (*d*) CP subunit β4 does not interact with UBLCP1. HEK293T cells were transfected with FLAG-β4. After 48 h, cell lysates were subjected to IP using anti-FLAG antibody. IP products were probed with indicated antibodies. (*e*) GST-UBLCP1 retrieved Rpt1, but not CP. GST-UBL^hRad23B^ and GST-UBLCP1 were purified from *E. coli*. Recombinant proteins were incubated with HEK293T lysates, respectively. GST-fusion proteins bound to proteasome complex were detected by western blotting with indicated antibodies.

We have previously reported the critical regulatory functions of the FCP/SCP family protein phosphatases in cell signalling [36,42]. To further study other small C-terminal domain phosphatases (SCPs), we have initiated a series of experiments looking for regulators and substrates of SCPs. We independently found the interaction of UBLCP1, a member of the SCP family, with the proteasome. During the course of our study, such interaction was also reported by Guo *et al.* [33]. Our further extensive analysis using immunoprecipitation-coupled mass spectrometry (IP-MS) revealed that the majority of RP subunits and two RP assembly chaperones, p28/gankyrin and PAAF1, were present in the anti-FLAG IP products from HEK293T cells stably expressing FLAG-UBLCP1 (table 1). Intriguingly, no CP subunits were retrieved. We further performed co-immunoprecipitation (co-IP) experiments to validate whether UBLCP1 binds to individual subunits of the proteasome. The ability of HA-tagged UBLCP1 or its phosphatase-dead mutant DDAA to co-precipitate with FLAG-tagged proteasome subunits was examined by IP-coupled western blotting. As shown in figure 1*a*, both UBLCP1 and DDAA were readily detected with immunoprecipitated RP subunits, Rpt1 and Rpn1, and chaperone PAAF1, but not with CP subunit β4 in HEK293T cells. Meanwhile, the direct interaction between UBLCP1 and proteasome subunits was determined by *in vitro* binding assay where only recombinant proteins were used. Recombinant UBLCP1 and DDAA proteins were expressed and purified from *E. coli* as glutathione *S*-transferase (GST) fusion proteins, while proteasome subunits were obtained from *in vitro* coupled transcription/translation in rabbit reticulocyte lysate. As seen in figure 1*b*, GST-UBLCP1 and GST-DDAA strongly interacted with FLAG-Rpn1, weakly with FLAG-tagged Rpt1, Rpt2, Rpt3, Rpt6 and PAAF1. FLAG-Smad5 was used as a negative control. These data suggest that UBLCP1 directly interacts with Rpn1, which is in agreement with Guo *et al.*'s finding [32,33].

**Table 1.** Mass spectrometry datasheet of UBLCP1 interacting proteins^a^.

no. peptide (IP: α-UBLCP1)	no. peptide (IP: α-IgG)	protein	alias	activity type
149	4	UBLCP1		
*19S regulatory particle*				
36	0	PSMC1	Rpt2	AAA ATPase
24	1	PSMD2	Rpn1	scaffold
23	1	PSMD6	Rpn7	
21	1	PSMC2	Rpt1	AAA ATPase
19	0	PSMD13	Rpn9	
18	1	PSMC5	Rpt6	AAA ATPase
8	1	PSMD7	Rpn8	
7	0	PSMC6	Rpt4	AAA ATPase
7	0	PSMC4	Rpt3	AAA ATPase
6	0	PSMD1	Rpn2	scaffold
6	1	PSMD11	Rpn6	
6	0	PSMD14	Rpn11	
6	0	PSMD8	Rpn12	
4	0	PSMD12	Rpn5	
4	0	PSMD3	Rpn3	
1	0	PSMD4	Rpn10	
1	1	ADRM1	Rpn13	
0	1	PSMC3	Rpt5	AAA ATPase
*19S assembly chaperone*				
12	0	PSMD10	p28/gankyrin	
8	0	PAAF1	Rpn14	
*20S core particle*				
0	2	PSMA1	α1	
0	1	PSMA2	α2	
1	2	PSMA3	α3	
0	1	PSMA4	α4	
0	2	PSMA5	α5 iso.1	
1	0	PSMA5	α5 iso.2	
1	0	PSMA6	α6	
0	1	PSMA7	α7	
1	1	PSMB1	β1	
0	2	PSMB4	β4	
1	0	PSMB6	β6	

^a^FLAG-UBLCP1 and associated proteins in lysates from HEK293T cell stably overexpressing FLAG-UBLCP1 were immunoprecipitated using anti-UBLCP1 antibody or control rabbit IgG. IP products were subjected to mass spectrometry analysis.

Furthermore, we performed endogenous co-IP by using UBLCP1 antibody. We observed an interaction between endogenous UBLCP1 and Rpn1 in HEK293T cells (figure 1*c*, lane 3), indicating that UBLCP1 interacts with Rpn1 under physiological conditions. In contrast, we found that UBLCP1 failed to interact with CP subunit β4 in HEK293T cells, even though β4 is associated with Rpn1 and α1–7 in the experiment (figure 1*d*, lane 3). Taken together, these results suggest that UBLCP1 selectively interacts with the RP through Rpn1.

Besides the 26S proteasome, there are considerable free RPs and CPs in the cell. Free RPs can be the assembly intermediates of the 26S proteasome or have non-proteolytic function in the nucleus, such as transcription [43]. In order to distinguish whether UBLCP1 binds to the 26S proteasome or free RP, we compared the proteasome binding ability of GST-UBLCP1 and GST-UBL^hRad23B^ to purified proteasomes. GST-UBL^hRad23B^ has high affinity to RP subunit Rpn10 [44], and GST-UBL^hRad23B^ pull-down was used as a routine method to purify proteasomes from mammalian cells. As seen in figure 1*e*, compared with GST-UBL^hRad23B^, GST-UBLCP1 retrieved comparable amounts of Rpt1, but profoundly less α1–7 subunits (figure 1*e*, lane 4). These data collectively show that, unlike hRad23B, UBLCP1 preferentially binds to RP but not the 26S proteasome.

### 3.2. UBLCP1 impairs proteasome assembly during the RP–CP association

Guo *et al.* [33] reported that UBLCP1 regulates RP–CP association. However, it is still unclear whether the UBLCP1 disrupts the assembly of the proteasome or promotes the disassembly of the proteasome. Our IP-MS experiment showed that proteasome assembly chaperones p28/gankyrin and PAAF1 were co-purified with UBLCP1 (table 1). Since assembly chaperones are absent in the mature proteasome, we speculate that UBLCP1 binds to Rpn1 during proteasome assembly. Previous reports show that the proteasome assembly is correlated with proteasome phosphorylation [45], which implies that the proteasome assembly could possibly be regulated by dephosphorylation by UBLCP1.

**Figure 2.**
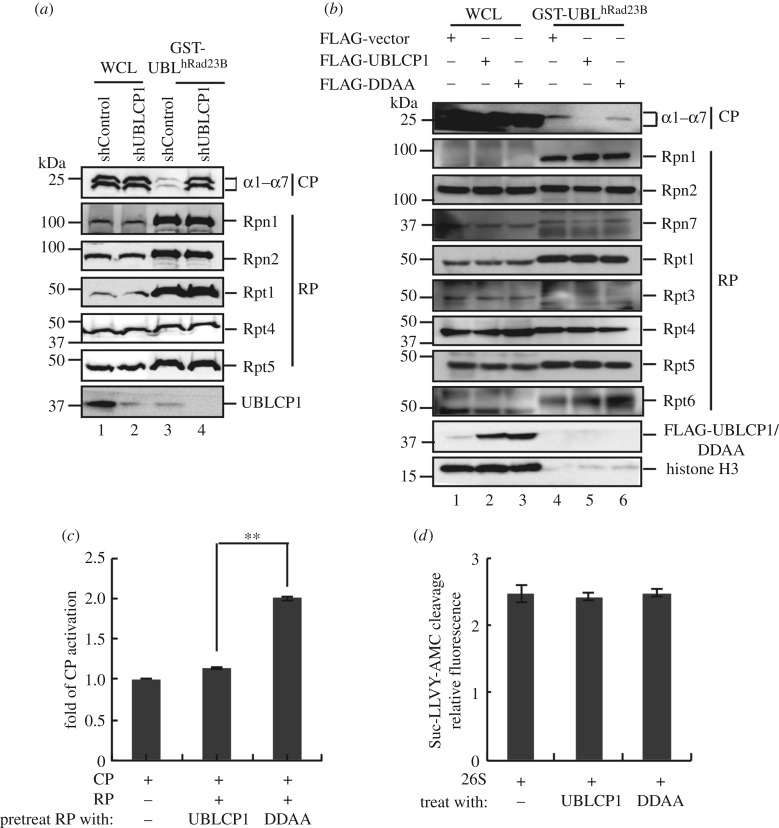
UBLCP1 impairs RP–CP association. (*a*) UBLCP1 knockdown enhances the 26S proteasome integrity. Proteasome integrity analysis was performed by GST-UBL^hRad23B^ pull-down methods in HEK293T stable cell lines stably expressing shUBLCP1 and shControl. (*b*) UBLCP1, but not DDAA, impairs the 26S proteasome integrity. Proteasome integrity analysis was performed by GST-UBL^hRad23B^ pull-down methods in HEK293T stable cell lines following western blotting with indicated proteasome subunit antibodies. (*c*) GST-UBLCP1 prevents RP from activating CP. Free RP was purified from HEK293T cells stably expressing HTBH-tagged Rpn11. Free CP was purified from HEK293T cells transfected with FLAG-tagged β4. RP was pretreated with GST-UBLCP1 and GST-DDAA, respectively. The proteasome was reconstituted by incubating CP with RPs. Reconstituted proteasome was incubated with Suc-LLVY-AMC substrate. AMC release was further detected at 460 nm after 380 nm excitation using a fluorescence plate reader. The values were normalized with the amount of CP. All data are the means (±s.e.) of three independent experiments performed in triplicate; **p < 0.01, paired Student's *t*-test. (*d*) GST-UBLCP1 does not affect the 26S proteasome activity. The 26S proteasome holoenzyme was purified from HEK293T cells stably expressing HTBH-tagged Rpn11, and then incubated with purified GST-UBLCP/DDAA *in vitro*. Then proteasome activity assay was performed as for (*c*).

To explore the function of UBLCP1 on proteasome integrity, we used GST-UBL^hRad23B^ as the bait to isolate the 26S proteasome. As the first step to assess the function of UBLCP1, we established HEK293T cells stably expressing UBLCP1-specific short-hairpin RNA (shUBLCP1). Notably, UBLCP1 knockdown could enhance the yield of CP subunits associated with GST-UBL^hRad23B^ (figure 2*a*, lane 3–4), while all the RP subunits tested had the same yields in GST-UBL^hRad23B^ pull-down products. Conversely, overexpression of UBLCP1, but not the DDAA mutant, significantly reduced the CP subunits retrieved by GST-UBL^hRad23B^ pull-down in HEK293T cells, while neither had effects on retrieved RP subunits (figure 2*b*). We further performed the reverse immunoprecipitation by using anti-20S antibody and found that less RP subunit was immunoprecipitated in UBLCP1 overexpressing cells (electronic supplementary material, figure S1*a*), which indicates that the RP–CP association can be disrupted by UBLCP1. Therefore, these results demonstrate that UBLCP1 impairs the integrity of 26S proteasome, yet appears to preserve the RP integrity.

We next used an *in vitro* reconstitution system to assess the efficiency of 26S proteasome assembly from RP and CP. Free RP, free CP and 26S proteasome were purified from HEK293T cells with UBLCP1 being depleted by *hSpCas9*-mediated knockout. We found that pre-incubation with recombinant GST-UBLCP1 disabled RP to bind and activate CP, while RP pretreated with phosphatase-dead GST-DDAA retained its ability to activate CP in degradation of artificial substrate Suc-LLVY-AMC (figure 2*c*). Intriguingly, incubating GST-UBLCP1 or GST-DDAA protein with proteasome holoenzyme had no effect on 26S proteasome activity towards Suc-LLVY-AMC (figure 2*d*). These results collectively suggest that UBLCP1 preferentially sequestrates RP from activating CP while leaving alone proteasome holoenzyme. Moreover, the phosphatase activity of UBLCP1 is required for the inhibitory function on proteasome assembly and activity.

### 3.3. UBLCP1 does not affect the RP integrity

**Figure 3.**
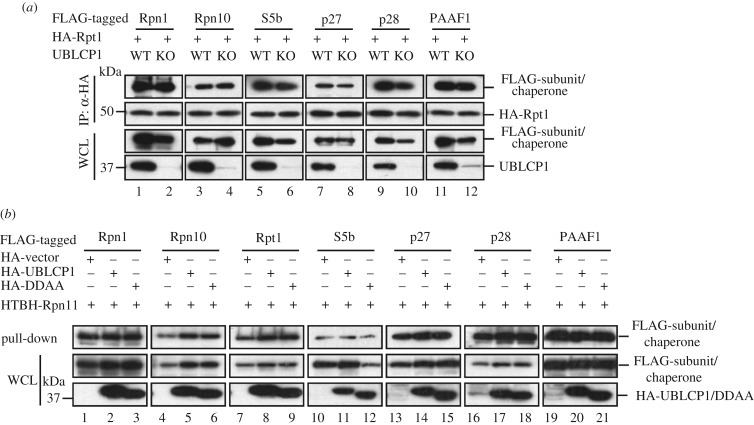
UBLCP1 does not affect RP integrity. (*a*) Loss of UBLCP1 does not affect chaperones and other subcomplexes binding to Rpn1-Rpt1-Rpt2-S5b subcomplex. HA-Rpt1 was co-transfected with FLAG-tagged RP assembly chaperones Rpn1 and Rpn10 into HEK293T cells with or without *hSpCas9*-mediated UBLCP1 knockout. Co-IP experiments were performed as previously. (*b*) UBLCP1 does not affect the chaperones–proteasome association. HA-tagged UBLCP1 or DDAA and FLAG-tagged proteasome assembly chaperones or proteasome subunits were co-transfected into HEK293T cells stably expressing HTBH-tagged Rpn11. Streptavidin–agarose pull-down products were detected by western blotting with indicated antibodies.

Since UBLCP1 binds to RP, we examined whether the effect of UBLCP1 on the RP–CP assembly could be attributed to its regulation on the RP integrity. We thus investigated whether UBLCP1 affects the interactions between the Rpn1-Rpt1-Rpt2-S5b subcomplex and other subcomplexes or chaperones in the RP. To this end, we performed co-IP experiments in HEK293T cells with *hSpCas9*-mediated knockout of the UBLCP1 gene. Loss of UBLCP1 did not affect the interaction between Rpt1 and Rpn1 or between Rpt1 and Rpn10 (figure 3*a*, lane 1–4). None of the interactions between Rpt1 and proteasome assembly chaperones, including S5b, p27, p28 and PAAF, were affected by UBLCP1 knockout (figure 3*a*, lane 5–12).

We also tested the effect of overexpressed UBLCP1 on the RP integrity, as assessed by examining the lid–base association in the RP. We established a HEK293T cell line that stably expresses HTBH-tagged Rpn11, a component in the lid of RP. HA-UBLCP1 or HA-DDAA was co-transfected with individual FLAG-tagged base subunits or RP assembly chaperones into the HEK293T-Rpn11 cells. Base subunits and RP assembly chaperones will be recovered in the products of streptavidin–agarose pull-down of Rpn11 [30]. As shown in figure 3*b*, overexpression of UBLCP1 or DDAA had no effects on the levels of chaperones S5b, p27, p28 and PAAF1 and proteasome subunits Rpn1, Rpn10 and Rpt1 retrieved on streptavidin–agarose beads. These results demonstrate that UBLCP1 and its phosphatase activity do not play a role in the lid–base association or the chaperones–proteasome association.

### 3.4. UBLCP1 blocks degradation of proteasome substrates

**Figure 4.**
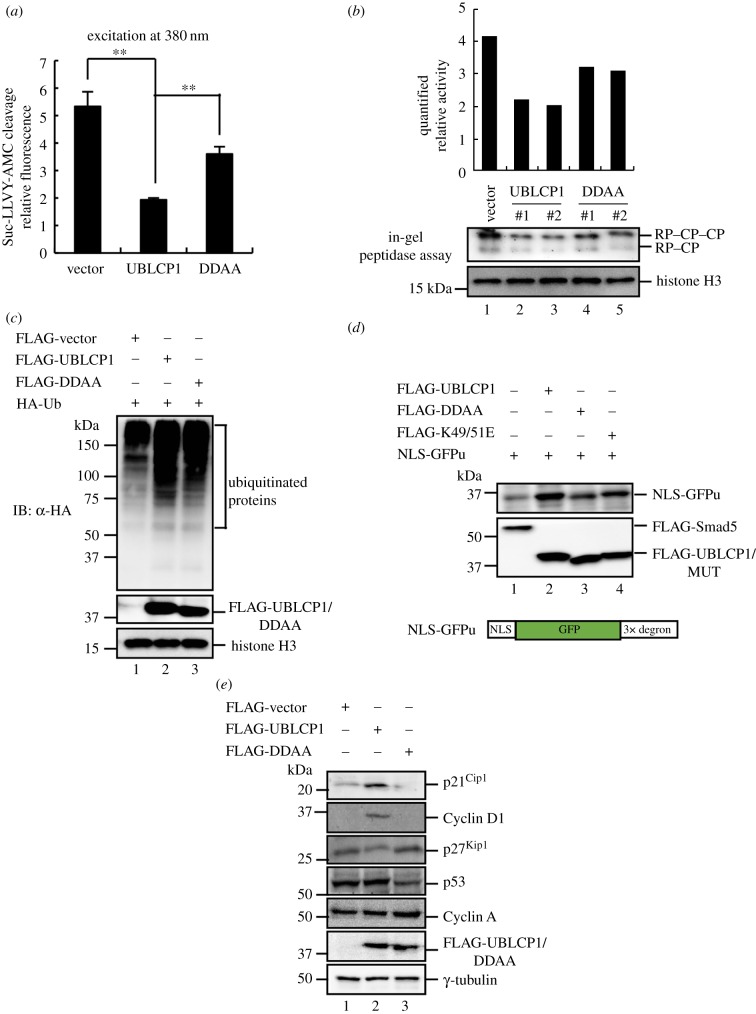
UBLCP1 blocks degradation of proteasome substrates. (*a*) UBLCP1 impairs the activity of the nuclear proteasome. Nuclear lysates were fractionated from HEK293T cells stably overexpressing UBLCP1 or DDAA. The lysates were incubated with Suc-LLVY-AMC substrate, and the proteasome activity assay was performed as in figure 2*c*. All data are the means (±s.e.) of three independent experiments performed in triplicate; **p < 0.01, paired Student's *t*-test. (*b*) UBLCP1 attenuates the activity of the nuclear proteasome in in-gel peptidase assays. The nuclear proteasome was purified by using GST-UBL^hRad23B^ pull-down and then subjected to native PAGE. The native PAGE was incubated with Suc-LLVY-AMC substrate and AMC release was visualized under UV light. The intensity of the bands was quantified with ImageJ. #1 and #2 indicate two individual stable cell lines. (*c*) UBLCP1 enhances accumulation of poly-ubiquitinated proteins in the nucleus. HA-Ubiquitin and FLAG-UBLCP1 or mutants were co-transfected into HEK293T cells. Nuclear lysates were directly subjected to western blotting with indicated antibodies. (*d*) UBLCP1 enhances the stability of NLS-GFPu *in vivo*. Transient overexpression of NLS-GFPu and FLAG-UBLCP1 or mutants in HEK293T cells. Cell lysates were directly subjected to western blotting with indicated antibodies. NLS, nuclear localization signal. GFPu, GFP fusions to a ubiquitin-proteasome CL1 degron. CL1 degron sequence, ACKNWFSSLSHFVIHL. (*e*) UBLCP1 enhances endogenous nuclear proteasomal substrate stability. FLAG-UBLCP1 or mutants were transfected into HEK293T cells, and cell lysates were subjected to western blotting with indicated antibodies.

Immunofluorescence staining of transfected HA-UBLCP1 in HeLa cells confirmed that UBLCP1 is a nuclear protein (electronic supplementary material, figure S1*b*), implying that UBLCP1 might regulate proteasome activity in the nucleus. We examined the nuclear proteasome activity by using the nuclear lysates fractionated from HEK293T cells stably overexpressing UBLCP1 or DDAA. UBLCP1 led to a significant reduction in proteasome activity towards Suc-LLVY-AMC *in vivo* (figure 4*a*). We further validated the physiological function of UBLCP1 by the loss-of-function strategy. Conversely, UBLCP1 knockdown by short interference RNA (siRNA) promotes Suc-LLVY-AMC cleavage by nuclear lysates fractionated from HEK293T cells (electronic supplementary material, figure S1*c*). In addition, UBLCP1 attenuated the nuclear proteasome activity using an in-gel peptidase assay (figure 4*b*, lane 2–3). These results suggest that UBLCP1 negatively regulates the proteasome activity in the nucleus.

Because the proteasome is responsible for degrading the majority of proteins in eukaryotic cells, we then tested whether UBLCP1 acts to enhance stability of proteasome substrates in the cell. Consistent with the fact that proteasome substrates are poly-ubiquitinated before degradation by the proteasome, we found overexpression of UBLCP1 significantly enhanced the accumulation of poly-ubiquitinated proteins in the nucleus (figure 4*c*). We also used an artificial nuclear proteasome substrate, NLS-GFPu, to assess the effect of co-transfected UBLCP1 or its mutants on proteasome substrates in HEK293T cells. NLS-GFPu was a labile protein when the proteasome was not attenuated (figure 4*d*, lane 1). Overexpression of UBLCP1, but not DDAA, significantly increased NLS-GFPu stability (figure 4*d*). Furthermore, we evaluated the stability of endogenous proteasome substrates such as p21^Cip1^ and Cyclin D1 in HEK293T cells. Overexpression of UBLCP1 considerably increased the stability of p21^Cip1^ and Cyclin D1, whereas overexpression of DDAA did not show this effect (figure 4*e*). However, we found some but not all proteasome substrates are stabilized in the same manner, perhaps due to differential subcellular localizations or unknown mechanisms. Taken together, all these results suggest that UBLCP1 downregulates the proteasome activity.

### 3.5. The UBL domain of UBLCP1 interacts with the distal C-terminal region of Rpn1

**Figure 5.**
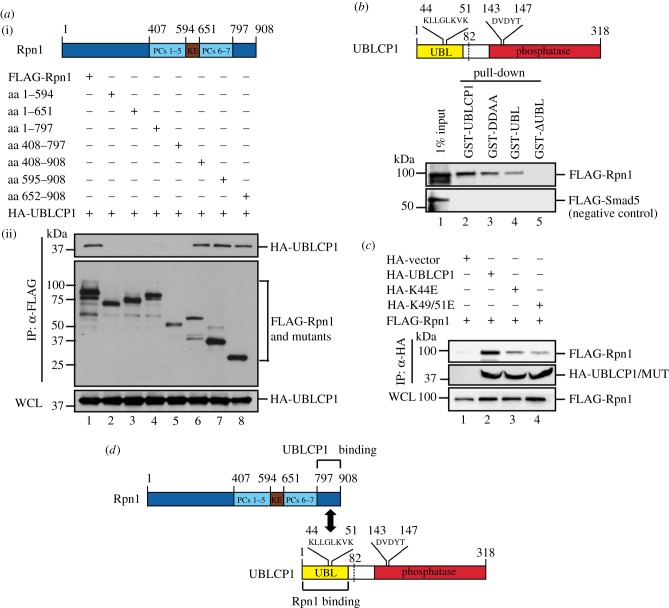
The UBL domain of UBLCP1 interacts with the distal C-terminal region of Rpn1. (*a*) UBLCP1 interacts with the C-terminal region of Rpn1. (i) Schematic of Rpn1. KE, motif rich in alternating lysine (K) and glutamate (E) residues. PCs, proteasome/cyclosome (PC) repeat. (ii) Rpn1 truncations were generated by PCR. HA-UBLCP1 was co-transfected with FLAG-Rpn1 or its individual truncation into HEK293T cells and co-immunoprecipitated by anti-FLAG antibody, followed by western blotting using indicated antibodies. WCL was shown. (*b*) The UBL domain of UBLCP1 mediates its binding to Rpn1. Schematic of UBLCP1 is shown at the top. UBL, ubiquitin-like domain. GST pull-down was performed by after co-incubation of *in vitro* translated FLAG-Rpn1 and a purified recombinant GST-fusion protein, GST-UBLCP1, GST-DDAA, GST-UBL or GST-ΔUBL, respectively. FLAG-Smad5 was used as negative control in the pull-down experiment. (*c*) K44E and K49/51E mutants of UBLCP1 have reduced binding to Rpn1. Co-IP of FLAG-Rpn1 and HA-UBLCP1 or HA-KE mutation (site 44 or sites 49/51) was carried out in HEK293T cells. UBLCP1-KE mutants, i.e. lysine (K) mutated to glutamic acid (E), were generated as previously. (*d*) Schematic of the interaction between UBL domain of UBLCP1 and C-terminal region of Rpn1.

To identify the structural features that determine the UBLCP1–Rpn1 interaction, we mapped the interaction domains in both proteins. To determine which Rpn1 regions bind to UBCLP1, we transfected HEK293T cells with UBLCP1 and a series of Rpn1 truncation mutants. The ability of Rpn1 mutants to co-immunoprecipitate with UBLCP1 was examined by IP-coupled western blotting. As shown in figure 5*a*, the truncated Rpn1 (aa 408–908, aa 595–908 and aa 652–908) mutants could pull-down HA-UBLCP1, whereas the truncated Rpn1 (aa 1–594, aa 1–651, aa 1–797 and aa 408–797) mutants were unable to bind to UBLCP1. These results provide strong evidence that the C-terminal region of Rpn1 (aa 798–908) is responsible for UBLCP1 binding.

Meanwhile, the Rpn1-binding domain on UBLCP1 was determined by direct *in vitro* binding assays. Purified recombinant GST-fusion proteins of UBLCP1, DDAA, UBL (aa 1–81) or ΔUBL (aa 82–318) were incubated with *in vitro* translated FLAG-tagged Rpn1. As shown in figure 5*b*, FLAG-Rpn1 strongly interacted with GST-UBL, but not with ΔUBL, indicating that the UBL domain of UBLCP1 is necessary and sufficient for Rpn1 binding. To further identify Rpn1-binding residues in the UBL domain of UBLCP1, we performed co-IP experiments in HEK293T cells. As Guo *et al.* [33] reported earlier, Lys^44^ of UBLCP1 mediates the interaction to Rpn1. Indeed, we confirmed that the UBLCP1 K44E mutant impaired the interaction between UBLCP1 and Rpn1 (figure 5*c*). Based on the study by Yun *et al.* [31], who reported the structure of the UBL domain of human UBLCP1 and suggested that the β3-α2 loop is responsible for Rpn1 binding, we noted that residues Lys^49^ and Lys^51^ are located in the unique β3-α2 loop, and are importantly conserved in UBLCP1 among different species and absent in other UBL-containing proteins. Thus, we also determined the critical role of Lys^49^ and Lys^51^ in UBLCP1–Rpn1 interaction (figure 5*c*). Compared to wild-type UBLCP1, the K49/51E mutant lost its inhibitory effect on nuclear proteasome activity (electronic supplementary material, figure S1*d*). Collectively, these data show that the UBL domain of UBLCP1 preferentially recognizes the C-terminal region of Rpn1 (figure 5*d*).

### 3.6. UBLCP1 dephosphorylates the RP subunit Rpt1

**Figure 6.**
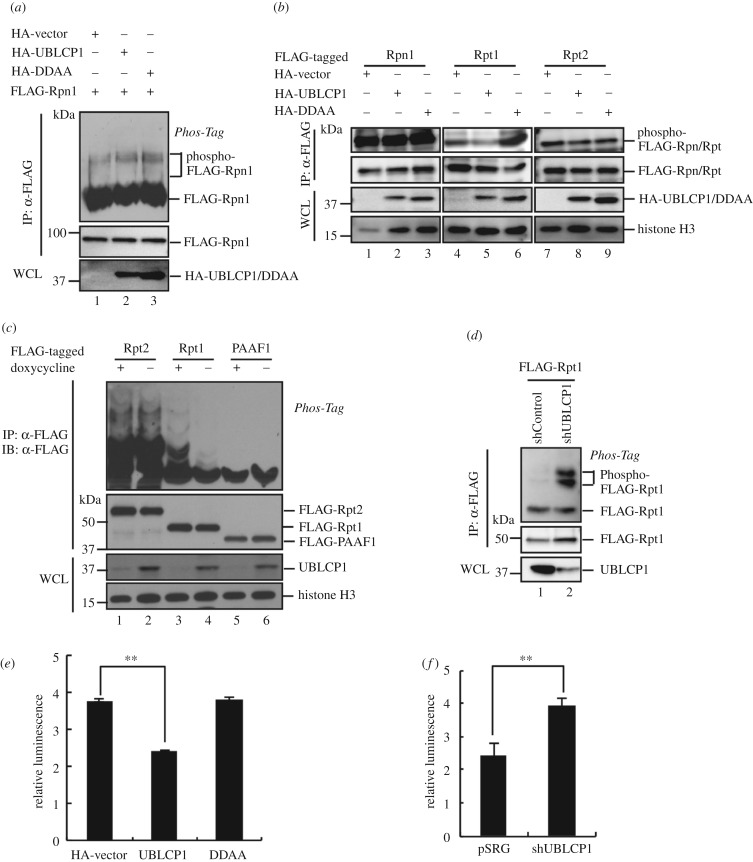
UBLCP1 dephosphorylates Rpt1. (*a*) UBLCP1 does not alter Rpn1 phosphorylation. HEK293T cells were transfected with FLAG-Rpn1 and HA-UBCLP1 or DDAA mutant. After 48 h, cell lysates were subjected to immunoprecipitation using anti-FLAG antibody. IP products were probed with indicated antibodies. (*b*) UBLCP1, but not DDAA mutant, dephosphorylates Rpt1. HEK293T cells were transfected with FLAG-tagged proteasome subunits and HA-UBCLP1 or DDAA mutant. After 48 h, cell lysates were subjected to immunoprecipitation using anti-FLAG antibody. IP products were analysed with indicated antibodies. Phosphorylation levels of FLAG-tagged Rpn1, Rpt1 and Rpt2 were evaluated with an anti-phosphoserine antibody. (*c*) UBLCP1 removes up-shifted Rpt1 bands on Phos-tag gel. Samples were prepared as (*a*) from HeLa Tet-Off cell line with inducible UBLCP1 expression when doxycycline was removed. IP products were resolved on Phos-tag gel and SDS-PAGE, and then analysed by western blotting with indicated antibodies. (*d*) shUBLCP1 enhances Rpt1 phosphorylation on Phos-tag gel. Samples were prepared as (*a*). IP products were resolved on Phos-tag gel and SDS-PAGE, and then analysed by western blotting with indicated antibodies. (*e*) UBLCP1, but not DDAA, impairs Rpt1 ATPase activity in HEK293T cells. FLAG-Rpt1 was co-transfected with HA-UBLCP1 or DDAA mutant. After 48 h, cell lysates were subjected to immunoprecipitation using anti-FLAG antibody. IP products were incubated with ATP at 37°C for 30 min. ADP production was measured by ADP-Glo assay. Luminescence was read using a luminometer. All data are the means (±s.e.) of three independent experiments performed in triplicate; **p < 0.01, paired Student's *t*-test. (*f*) UBLCP1 knockdown enhances Rpt1 ATPase activity in HEK293T cells. FLAG-Rpt1 was transfected into shUBLCP1 and shControl cell lines. Samples were prepared and their ATPase activities were measured as in (*e*).

To identify the potential substrates of UBLCP1, we first examined whether Rpn1 is a direct substrate of UBLCP1 because of their direct interaction. Rpn1 was detected as a phospho-protein by using the Phos-tag method. Notably, we failed to observe any difference in Rpn1 phosphorylation in the presence or absence of overexpressed UBLCP1 (figure 6*a*). This negative result was not attributed to lack of phosphatase activity, as we observed that GST-UBLCP1 had intrinsic phosphatase activity. Purified GST-UBLCP1, but not the catalytically inactive mutant DDAA, could dephosphorylate the substrate *p*-nitrophenyl phosphate (*p*-NPP) (electronic supplementary material, figure S1*e*). To this end, we hypothesized that Rpn1 may serve as a platform for UBLCP1 targeting to the substrate(s). To identify the *bona fide* phosphatase substrate(s) of UBLCP1, we performed the dephosphorylation screen on Rpn1 and all Rpt subunits. As shown in figure 6*b*, UBLCP1 attenuated the phosphorylation of Rpt1 in HEK293T cells (lane 5), whereas DDAA appeared to enhance Rpt1 phosphorylation (figure 6*b*, lane 6). However, both of UBLCP1 and DDAA had no effect on the phosphorylation of Rpn1 (figure 6*b*, lane 1–3), Rpt2 (figure 6*b*, lane 7–9) and Rpt3–6 (data not shown).

The UBLCP1 activity on Rpt1 was further confirmed by using the Tet-Off inducible expression system in HeLa cells. The up-shifted bands of Rpt1 (figure 6*c*, lane 3), not Rpt2 (figure 6*c*, lane 1–2), were markedly decreased by the induced expression of UBLCP1 (when doxycycline was removed) in Phos-tag gel (figure 6*c,* lane 4), indicating that the phosphatase activity of UBLCP1 was specific towards Rpt1, but not Rpt2. In contrast, no up-shifted band of PAAF1, a proteasome-dedicated chaperone, was found in the Phos-tag gel regardless UBLCP1 status (figure 6*c*, lane 5–6). These results suggest that Rpt1, but not other subunits, is dephosphorylated by UBLCP1 in HeLa cells.

To further evaluate the role of endogenous UBLCP1, we determined the effect of UBLCP1 depletion on Rpt1 phosphorylation. We found that Rpt1 phosphorylation was significantly enhanced in HEK293T cells stably expressing shUBLCP1 (figure 6*d*). Taken together, these results suggest that the RP subunit Rpt1 is a substrate of phosphatase UBLCP1. We were next curious about the site(s) dephosphorylated by UBLCP1 in Rpt1. We generated a series of truncation mutants and serine/threonine point mutants of Rpt1. However, UBLCP1 was able to dephosphorylate each of these truncations or point mutants (electronic supplementary material, figure S2*a,b*). We speculate that there are multiple phosphorylation sites on Rpt1.

### 3.7. UBLCP1 impairs the ATPase activity of Rpt1

Having found that UBLCP1 dephosphorylates Rpt1 and impairs proteasome assembly, we reasoned that there could be a connection between Rpt1 phosphorylation and proteasome assembly. Previous studies have shown that phosphorylation of proteasome ATPase subunits correlates with proteasome assembly [45]. Furthermore, ATP binding and hydrolysis play vital roles in proteasome assembly [46,47]. Thus, we proposed that the effect of UBLCP1 on Rpt1 dephosphorylation would directly regulate Rpt1 ATPase activity and proteasome assembly. We purified FLAG-tagged Rpt1 from HEK293T cells stably overexpressing UBLCP1 or DDAA and performed ATPase activity assay. As shown in figure 6*e*, overexpression of UBCLP1, but not the DDAA mutant, inhibited Rpt1 ATPase activity. Consistently, ATP hydrolysis activity of FLAG-Rpt1 was significantly enhanced upon UBLCP1 knockdown in HEK293T cells (figure 6*f*). Altogether, these results collectively prove that UBLCP1 negatively regulates the ATPase activity of Rpt1.

### 3.8. Rpn1 is required for UBLCP1-mediated Rpt1 dephosphorylation and proteasome regulation

**Figure 7.**
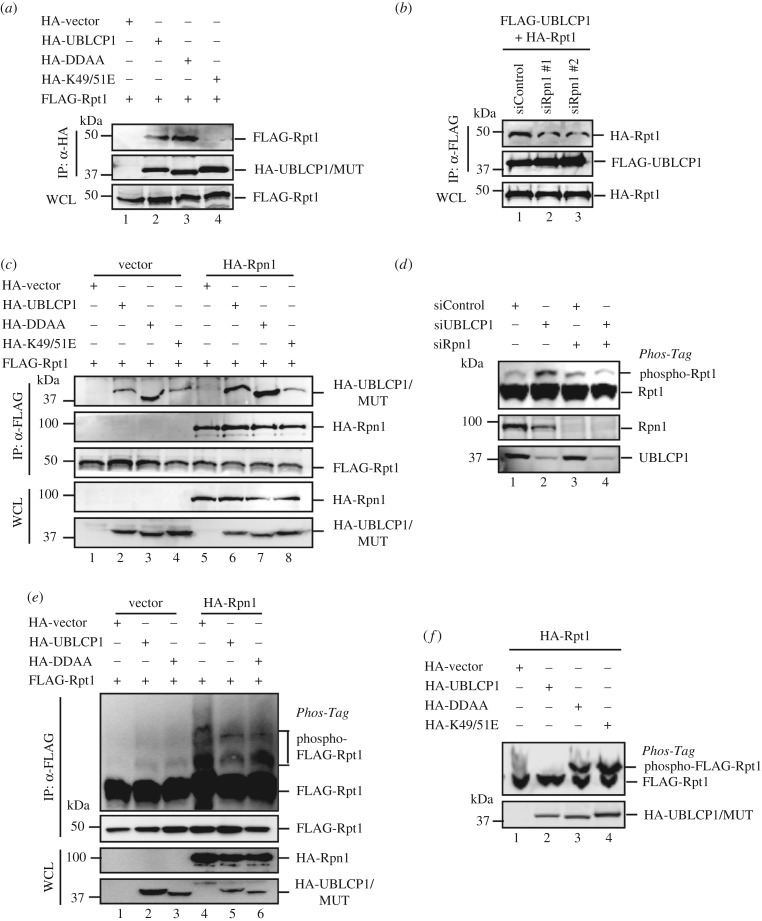
UBLCP1 regulates the proteasome in an Rpn1-dependent manner. (*a*) UBLCP1-K49/51E mutants fail to associate with Rpt1. FLAG-Rpt1 was co-transfected with HA-tagged wild-type or mutated UBLCP1. After 48 h, cell lysates were subjected to immunoprecipitation using anti-HA antibody and analysed by western blotting with indicated antibodies. (*b*) Rpn1 knockdown impairs the UBLCP1–Rpt1 association. siRpn1 was co-transfected with FLAG-UBLCP1 and HA-tagged proteasome subunits. After 48 h, cell lysates were subjected to immunoprecipitation using anti-FLAG antibody. IP products were probed with indicated antibodies. #1 and #2 indicate two individual short interference RNAs targeting Rpn1. (*c*) Rpn1 enhances the interaction between UBLCP1 and Rpt1. FLAG-Rpt1 and HA-UBLCP1/mutants were co-transfected with or without HA-Rpn1 in HEK293T cells. After 48 h, cell lysates were subjected to immunoprecipitation using anti-FLAG antibody. IP products were analysed by western blotting with indicated antibodies. (*d*) Rpn1 knockdown disables UBLCP1 to dephosphorylate Rpt1. siUBLCP1 was co-transfected with or without siRpn1 in HEK293T cells. After 36 h, cell lysates were resolved on Phos-tag gel and SDS-PAGE, and then analysed by western blotting with indicated antibodies. (*e*) Rpn1 enhances Rpt1 phosphorylation. FLAG-Rpt1 and HA-UBLCP1/mutants were co-transfected with or without HA-Rpn1 in HEK293T cells. After 36 h, cell lysates were subjected to immunoprecipitation using anti-FLAG antibody. IP products were resolved on Phos-tag gel and SDS-PAGE, and then analysed by western blotting with indicated antibodies. (*f*) UBLCP1 with K49/51E mutation loses its phosphatase activity towards Rpt1. FLAG-Rpt1 and HA-UBLCP1 mutant were co-transfected in HEK293T cells. After 36 h, cell lysates were subjected to immunoprecipitation using anti-FLAG antibody. IP products were resolved on Phos-tag gel and SDS-PAGE, and then analysed by western blotting with indicated antibodies.

One of the Rpn1 functions is to recruit multiple proteasome-interacting proteins (PIPs) [48]. Since both UBCLP1 and Rpt1 directly interact with Rpn1, we sought to examine whether UBLCP1 forms a larger complex with Rpn1-Rpt1. As shown in figure 7*a*, both UBLCP1 wild-type and the catalytically inactive mutant DDAA could interact with Rpt1. Notably, the UBLCP1-K49/51E mutant, which lost the ability to bind Rpn1 (figure 5*c*), also failed to associate with Rpt1 in the co-IP experiment (figure 7*a*, lane 4). Similarly, we observed that knockdown of Rpn1 by siRNA significantly reduced the UBLCP1–Rpt1 interaction in co-IP experiments (figure 7*b*, lane 2–3). These results suggest that the UBLCP1–Rpt1 association is likely mediated by Rpn1. Lastly, overexpression of Rpn1 enhanced the Rpt1–UBLCP1 interaction (figure 7*c*, lane 6–7). Accordingly, the UBLCP1-K49/51E mutant showed no increased affinity for Rpt1 even if Rpn1 was overexpressed (lane 4, 8). These results convincingly suggest that Rpn1 is essential in targeting UBLCP1 to Rpt1.

Having established the essential bridging role of Rpn1 in the UBLCP1–Rpt1 interaction, we sought to determine whether Rpn1 regulates the function of UBLCP1 on Rpt1 phosphorylation and proteasome activity. While siUBLCP1 increased the phosphorylation of Rpt1, depletion of Rpn1 abolished the effect of siUBLCP1 on Rpt1 phosphorylation (figure 7*d*, lane 3–4). We also examined the effect of Rpn1 overexpression on Rpt1 phosphorylation. Interestingly, Rpn1 overexpression enhanced Rpt1 phosphorylation (figure 7*e*, lane 4–6), probably through removal of UBLCP1 from Rpt1. Although the phosphatase activity of UBLCP1-K49/51E is not impaired in comparison with wild-type UBLCP1 *in vitro* (electronic supplementary material, figure S1*e*), UBLCP1-K49/51E failed to decrease the intensity of the up-shifted band of Rpt1 on Phos-tag gel (figure 7*f*, lane 4), further supporting the notion that Rpn1 directs UBLCP1 to the proteasome to dephosphorylate Rpt1. Furthermore, UBLCP1-K49/51E failed to affect proteasome activity on hydrolysis of Suc-LLVY-AMC (electronic supplementary material, figure S1*d*), and had little effect on NLS-GFPu degradation (figure 4*d*, lane 4). Taken together, these results collectively demonstrate that phospho-regulation of the proteasome by UBLCP1 depends on Rpn1.

## 4. Discussion

The UPS activity is regulated by transcription and posttranslational modification of the proteasome subunits. Phospho-regulation of the proteasome by UBLCP1 was reported before [33], yet its detailed mechanism is still not thoroughly elucidated. In an effort to study the SCP/FCP phosphatase family, we independently found UBLCP1 interacts with Rpn1 in the RP. Furthermore, we show that UBLCP1 dephosphorylates Rpt1 by interacting with the C-terminus of Rpn1. UBLCP1 negatively regulates the ATPase activity of Rpt1, the 26S proteasome assembly (i.e. the RP–CP association) and proteasome activity. Our work may provide insights into the understanding of other UBL-containing proteins acting in a similar way.

A previous report showed the residue Lys^44^ of human UBLCP1 is conserved through evolution and is essential for proteasome binding [33]. Our work further shows that the K49/51E mutation in UBLCP1 abolishes the UBLCP1–Rpn1 interaction (figure 5*c*), indicating that Lys^49^ and Lys^51^ in the UBL of human UBLCP1 are required for Rpn1 binding. Taken together, these results demonstrate that Lys^44^, Lys^49^ and Lys^51^ of UBL are required for Rpn1 interaction. On the other hand, the UBL domain of UBLCP1 specifically recognizes the C-terminal region of Rpn1. With the benefit of the cryo-EM revolution, several groups have reported the atomic model of yeast and human Rpn1 [49,50]. In yeast and human proteasomes, the C-terminal region of Rpn1 is located in the RP–CP interface. Moreover, the C-terminal region of Rpn1 is in proximity to the Rpn1–Rpt1 interface (electronic supplementary material, figure S3*a*), which aligns with our finding that UBLCP1 dephosphorylates Rpt1 by docking on the C-terminal region of Rpn1. In yeast and human cells, the proteasomal deubiquitinase Ubp6/USP14 was recently shown to bind also to Rpt1 [49–51]. The UBL domain of Ubp6/USP14 binds to the toroid domain of Rpn1, while the catalytic domain of Ubp6/USP14 binds to the OB domain of Rpt1, which is similar to the binding feature of UBLCP1. Moreover, the UBL domain of UBLCP1 and that of USP14 share the most similar sequence among UBL-containing proteins (electronic supplementary material, figure S3*b*), implying the evolutionary relationship between the two enzymes. We also investigated whether UBLCP1 impairs the binding ability of USP14 to Rpn1. As seen in the electronic supplementary material, figure S3*c*, UBLCP1 and USP14 competing assay shows no conflict in binding to Rpn1. Combining this with the fact that UBLCP1 is involved in proteasome assembly while USP14 binds to proteasome holoenzyme to remove the ubiquitin chain [8,52], we speculate that UBLCP1 and USP14 bind to the proteasome at different time windows.

**Figure 8.**
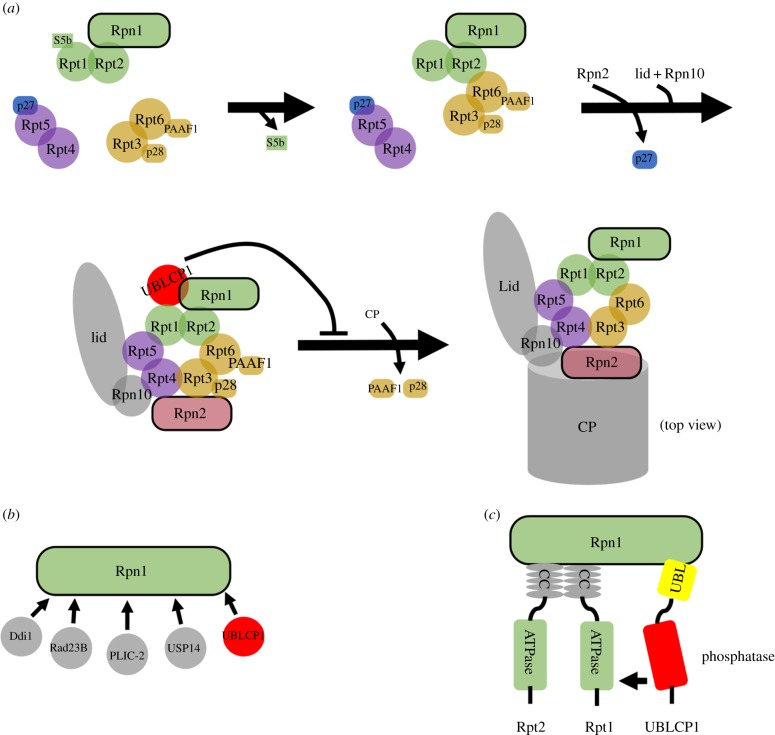
A working model for the role of UBLCP1 in 26S proteasome assembly. (*a*) A working model for proteasome regulation by UBLCP1. UBLCP1 selectively binds to RP precursor containing p28 and PAAF1. Rpn1 recruits UBLCP1 to dephosphorylate Rpt1. As a result, UBLCP1 impairs the RP–CP association, but preserves RP integrity. Arrangement of coloured proteasome base subunits is viewed from the top. (*b*) Schematic of reported Rpn1-interacting proteins. (*c*) A working model for UBLCP1 as a phosphatase of Rpt1. UBLCP1 dephosphorylates Rpt1 by interacting with the C-terminal of Rpn1. CC, coiled-coil.

The stepwise association and disassociation of proteasome-dedicated chaperones are required for correct and rapid assembly of the proteasome [53]. In mammalian cells, p28/gankyrin and PAAF1 likely interact with the base throughout the assembly of the RP, while S5b and p27 are released during the assembly of the RP [14]. Mass spectrometry analysis of UBLCP1-associated proteins revealed that UBLCP1 interacts with two proteasome-dedicated chaperones, p28/gankyrin and PAAF1, in HEK293T cells. Moreover, immunoprecipitated products retrieved by anti-UBLCP1 antibody are enriched in the precursor subcomplex Rpn1-Rpt1-Rpt2 and p28/gankyrin-Rpt3-Rpt6-PAAF1, which are the assembly intermediates of the RP base [2]. Furthermore, there is no evidence that UBLCP1 interacts with CP and two other proteasome-dedicated chaperones, S5b and p27. These data collectively imply that phosphatase UBLCP1 binds to the RP, to inhibit the 26S proteasome assembly (figure 8*a*).

Rpt1 is one of the six human proteasome ATPase subunits. Compared with other ATPases, Rpt1 has the largest interaction surface with the CP when hydrolysing ATP [16,54]. Furthermore, Rpt1 with ATP binding mutations is unable to be incorporated into the 26S proteasome, demonstrating that the ATPase activity of Rpt1 is required for the RP–CP association [46,47]. Remarkably, we also found that UBLCP1 impairs Rpt1 ATPase activity (figure 6*e,f*). Therefore, we speculate that the correlation between proteasome assembly and Rpt1 phosphorylation may be conferred by enhanced Rpt1 ATPase activity.

Rpn1 was reported as a scaffold to coordinate the ubiquitin processing factors at the proteasome (figure 8*b*) [48,55]. The UBL/UBA proteins, PLIC-2, Rad23B and Ddi1, function as shuttle receptors for ubiquitinated proteins. The UBL domain binds to Rpn1 while the UBA domain binds to ubiquitin, whereby they escort the ubiquitinated proteins to the proteasome for degradation [56]. Likewise, UBLCP1 binds to the proteasome through Rpn1 (figure 8*c*). Moreover, Rpn1 is a prerequisite for the UBLCP1 to dephosphorylate Rpt1 and regulate the proteasome. Intriguingly, we also find that Rpn1 overexpression enhances Rpt1 phosphorylation (figure 7*e*). It is tempting to speculate that Rpn1 can also recruit kinases to phosphorylate Rpt1. Rpn1 is probably the hub to coordinate phospho-regulation of the proteasome.

USP14 inhibitors lead to enhancement of the proteasome activity [57]. Similar to USP14, UBLCP1 inhibition by chemical inhibitors might also provide a clue to identify novel proteasome activators. Rabeprazole was reported as an SCP1 inhibitor by binding to the hydrophobic pocket at the CTD phosphatase domain [58]. Our work shows rabeprazole also has the ability to inhibit UBLCP1's effect on Rpt1 dephosphorylation *in vivo* (electronic supplementary material, figure S3*d*). He *et al.* [59] reported a UBLCP1-specific inhibitor compound 13 with IC_50_ of 1.0 µM. It will be interesting to test Rpt1 phosphorylation and proteasome function with this inhibitor in the future.

To accommodate the fast growth rates, cancer cells have a high level of protein generation and protein degradation. By adopting this feature, proteasome inhibitors such as bortezomib have been used as drugs to combat cancer by perturbing the proteolysis [60,61]. Any hypoactive effects on the proteasome, such as proteasomal gene mutation, will sensitize the effect of proteasome inhibitor. One recent study has identified that partial loss of Rpt1 brings a greater sensitivity to further suppression of Rpt1 in cancer cells [62], indicating Rpt1 is a putative cancer drug target. We find that overexpression of UBLCP1, but not DDAA, significantly impairs the viability of the SKOV3 ovarian cancer cell line, harbouring partial Rpt1 copy number loss (data not shown). On the contrary, UBLCP1 does not impair the viability of the A2780 ovarian cancer cell line, harbouring an intact Rpt1 copy number. We speculate that the anti-tumour effect of UBLCP1 in SKOV3 cells is based on Rpt1 inhibition. This data provides many clues to re-evaluate proteasome-inhibition-based drug design. In this study, we elucidate the basic mechanism of proteasome regulation by UBLCP1. However, more efforts are needed to answer some meaningful questions such as the upstream signal of Rpt1 phosphorylation and the regulator of UBLCP1.

## Supplementary Material

Supplementary material
