# Supplementary material for: Phosphatase UBLCP1 controls proteasome assembly
Source: Open Biol. 2017 May 24;7(5):170042. doi: 10.1098/rsob.170042 (PMC5451543; doi:10.1098/rsob.170042)
Supplement: Supplementary material [file rsob170042supp1.pdf]

Supplementary material

Figure S1. (*a*) UBLCP1, but not DDAA, abrogates the RP-CP association. HEK293T cells were collected, and cell lysates were subjected to immunoprecipitation using anti-α1-7 (20S) antibody. IP products and whole cell lysates were probed with indicated antibodies. (*b*) HA-UBLCP1 localizes in nucleus. HeLa cells transfected with HA-UBLCP1 were subjected to immunofluorescence analysis with anti-HA antibody. Nuclei were visualized by DNA staining with DAPI. (*c*) UBLCP1 knockdown enhances nuclear proteasome activity. Nuclear proteasome activity assay was performed as described in figure 4*a*. All data are the means (±s.e.) of three independent experiments performed in triplicates. *, $p<0.05$, paired Student's *t*-test. (*d*) UBLCP1 with the K49/51E mutations fails to regulate proteasome activity. Nuclear proteasome activity assay was performed as described in figure 4*a*. All data are the means (±s.e.) of three independent experiments performed in triplicates. *, $p<0.05$, paired Student's *t*-test. (*e*) Recombinant UBLCP1 and the K49/51E mutant, but not catalytically inactive DDAA mutant, possess phosphatase activity. Recombinant GST, GST-UBLCP1, GST-DDAA and GST-K49/51E proteins were purified from *E. coli*. *p*-nitrophenyl phosphate (*p*-NPP) was incubated with 1 μg of each protein. Absorbance at 405 nm was measured by a multi-well plate reader. All data are the means (±s.e.) of three independent experiments performed in triplicates.

Figure S2. (*a*) Rpt1 truncations are able to be dephosphorylated by UBLCP1. Rpt1 truncations were generated by PCR. FLAG-tagged Rpt1 truncations were co-transfected with HA-UBLCP1 or DDAA into HEK293T cells. The cell lysates were resolved on Phos-tag gel and SDS-PAGE, and then analyzed by Western blotting with indicated antibodies. (*b*) Rpt1 point mutants are able to be dephosphorylated by UBLCP1. Rpt1 point mutants were generated by PCR. FLAG-tagged Rpt1 point mutants were co-transfected with HA-UBLCP1 or DDAA into HEK293T cells. The cell lysates were resolved on Phos-tag gel and SDS-PAGE, and then analyzed by Western blotting with indicated antibodies. The serine/threonine residues were mutated to alanine in all the point mutants.

Figure S3. (*a*) Model of interface between Rpn1, Rpt1 and CP. Yeast proteasome model was retrieved with PDB ID: 5A5B. Images were generated by UCSF Chimera. Red, Rpn1; Green, Rpt1; Cyan, Rpt2; Light grey, CP; Yellow, C-terminal domain of Rpn1. (*b*) Phylogenetic analysis of UBL-containing proteins. Indicated protein sequences were collected from NCBI reference sequence library and multi-alignment was performed by using CLUSTALW. The phylogenetic tree was generated by using the maximum composite likelihood method (MEGA4). (*c*) UBLCP1 does not compete with USP14 for Rpn1 binding. HA-Rpn1 and FLAG-Rad23B or FLAG-USP14 were co-transfected into HEK293T cells with or without MYC-UBLCP1. 48 h later, cell lysates were subjected to immunoprecipitation using anti-HA antibody. IP products were probed with indicated antibodies. (*d*) Rabeprazole abolishes UBLCP1's phosphatase activity toward Rpt1. HEK293T was transfected as indicated. 12 h before harvesting, the cells were treated with Rabeprazole with the concentration of 50 μM and 100 μM, respectively. Cell lysates were resolved on Phos-tag gel and SDS-PAGE, and then proceed to Western blotting with indicated antibodies.

Figure S1

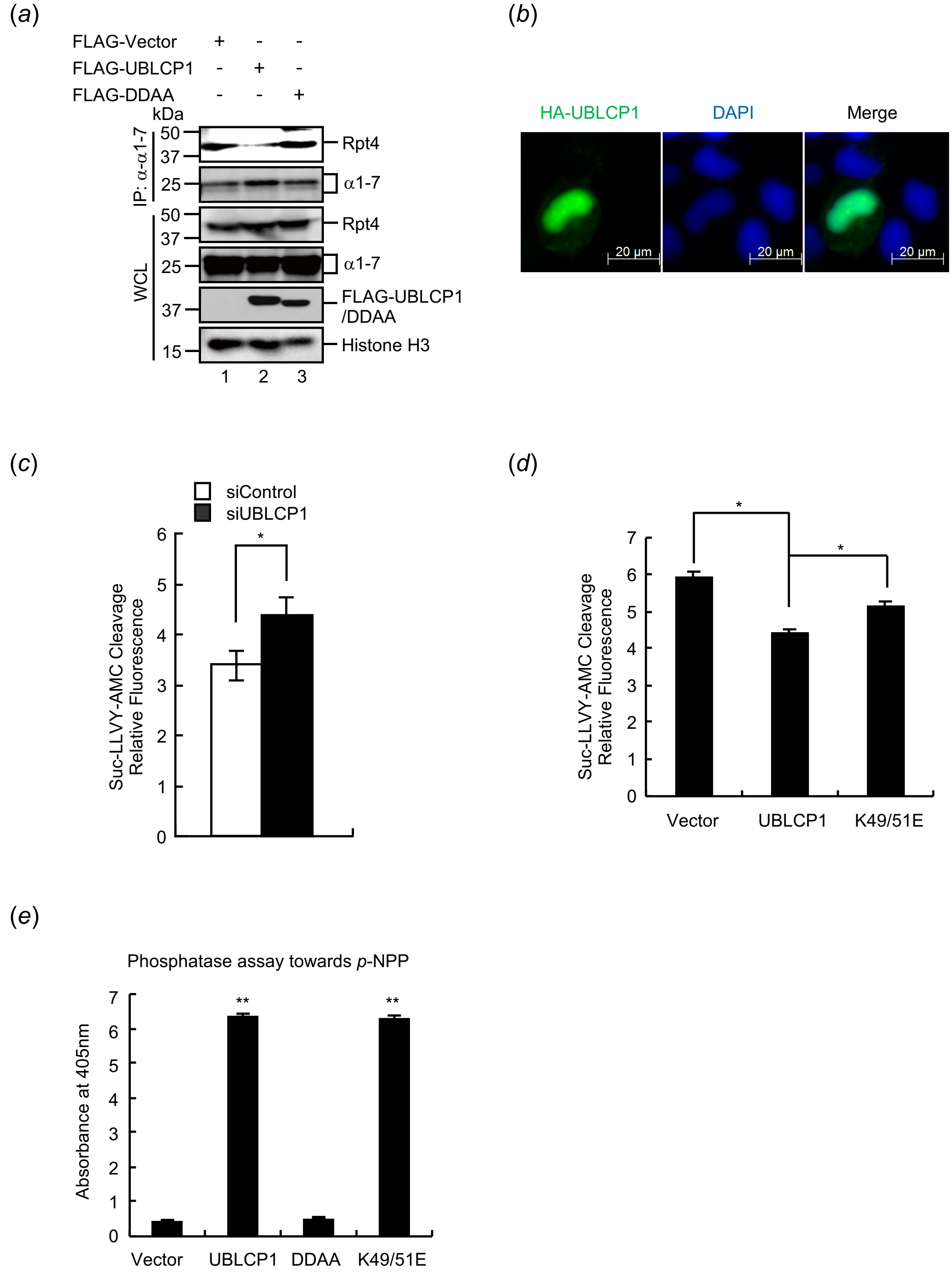
(a)
FLAG-Vector + - -
FLAG-UBLCP1 - + -
FLAG-DDAA - - +
kDa
IP: α-α1-7
WCL
50
37
25
15
Rpt4
α1-7
FLAG-UBLCP1 /DDAA
Histone H3
1 2 3
(b)
HA-UBLCP1
DAPI
Merge
20 μm
(c)
siControl
siUBLCP1
Suc-LLVY-AMC Cleavage Relative Fluorescence
*
(d)
Suc-LLVY-AMC Cleavage Relative Fluorescence
*
Vector
UBLCP1
K49/51E
(e)
Phosphatase assay towards p-NPP
Absorbance at 405nm
**
Vector
UBLCP1
DDAA
K49/51E

Figure S2

(*a*)

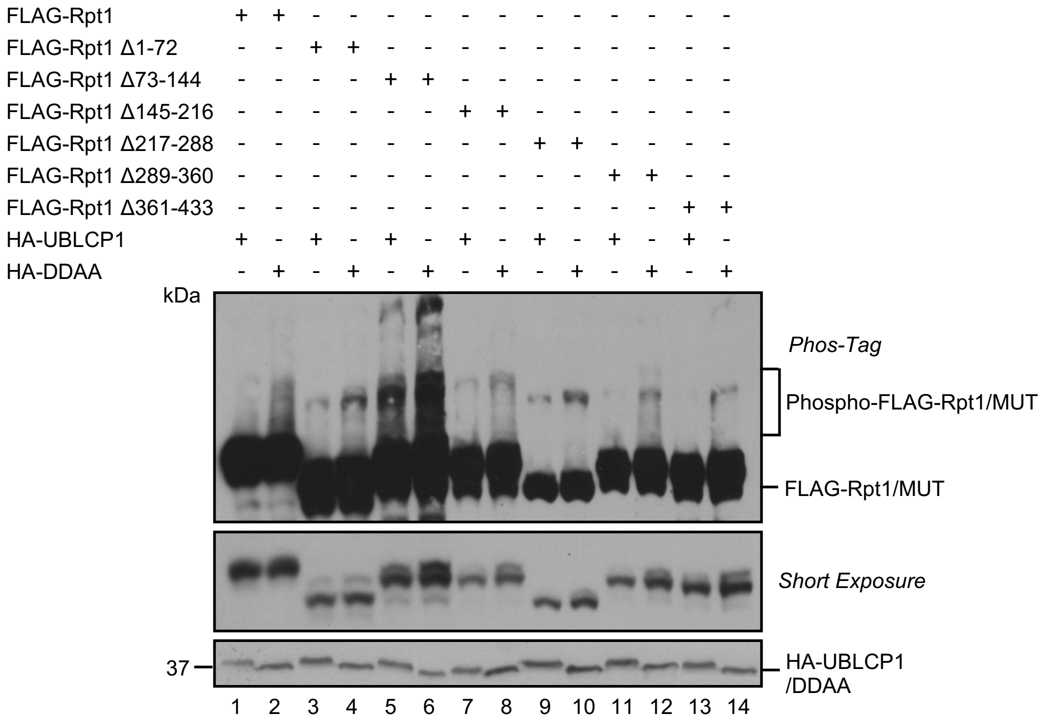

FLAG-Rpt1
FLAG-Rpt1 Δ1-72
FLAG-Rpt1 Δ73-144
FLAG-Rpt1 Δ145-216
FLAG-Rpt1 Δ217-288
FLAG-Rpt1 Δ289-360
FLAG-Rpt1 Δ361-433
HA-UBLCP1
HA-DDAA
kDa
Phos-Tag
Phospho-FLAG-Rpt1/MUT
FLAG-Rpt1/MUT
Short Exposure
37
HA-UBLCP1 /DDAA
1 2 3 4 5 6 7 8 9 10 11 12 13 14

(*b*)

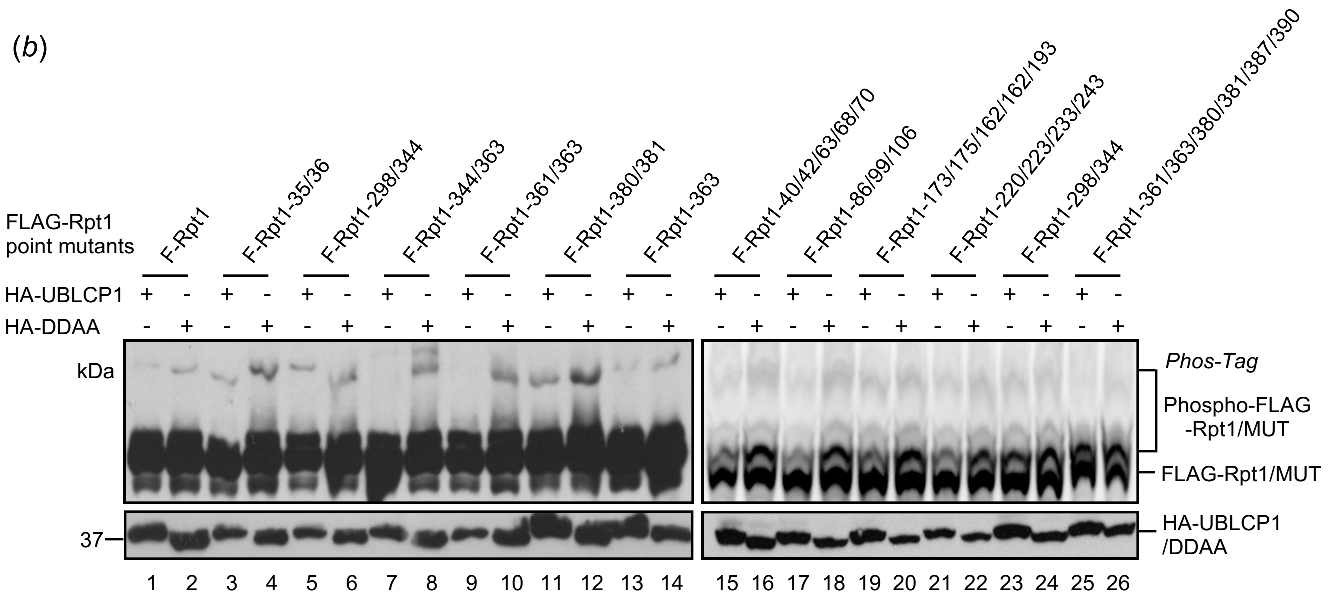

FLAG-Rpt1 point mutants
F-Rpt1
F-Rpt1-35/36
F-Rpt1-298/344
F-Rpt1-344/363
F-Rpt1-361/363
F-Rpt1-380/381
F-Rpt1-363
F-Rpt1-40/42/63/68/70
F-Rpt1-86/99/106
F-Rpt1-173/175/162/162/193
F-Rpt1-220/223/233/243
F-Rpt1-298/344
F-Rpt1-361/363/380/381/387/390
HA-UBLCP1
HA-DDAA
kDa
Phos-Tag
Phospho-FLAG -Rpt1/MUT
FLAG-Rpt1/MUT
37
HA-UBLCP1 /DDAA
1 2 3 4 5 6 7 8 9 10 11 12 13 14 15 16 17 18 19 20 21 22 23 24 25 26

Figure S3

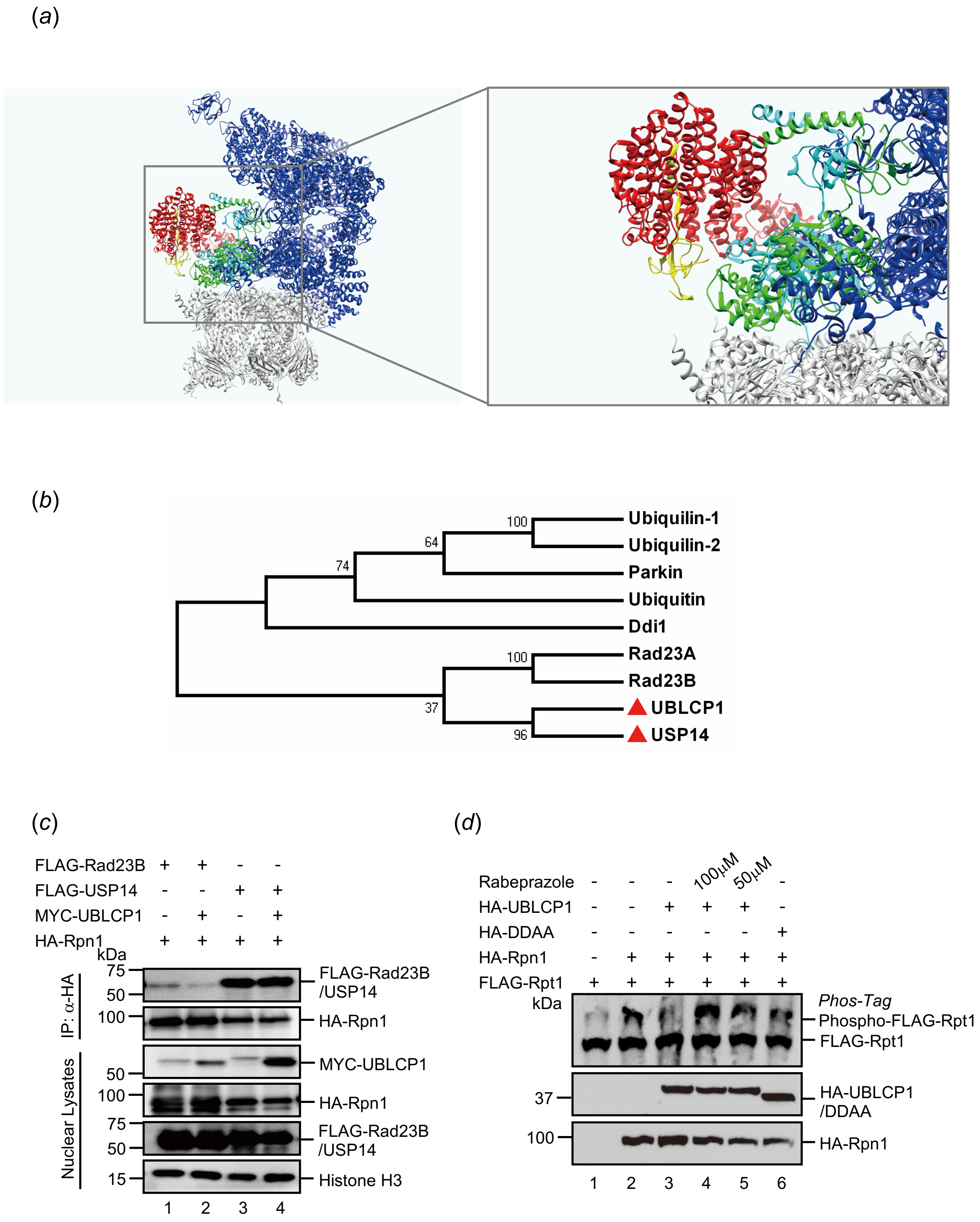

(a)
(b)
Ubiquilin-1
Ubiquilin-2
Parkin
Ubiquitin
Ddi1
Rad23A
Rad23B
UBLCP1
USP14
100
64
74
100
37
96
(c)
FLAG-Rad23B + + - -
FLAG-USP14 - - + +
MYC-UBLCP1 - + - +
HA-Rpn1 + + + +
kDa
IP: α-HA
Nuclear Lysates
75
50
100
50
100
75
50
15
FLAG-Rad23B /USP14
HA-Rpn1
MYC-UBLCP1
HA-Rpn1
FLAG-Rad23B /USP14
Histone H3
1 2 3 4
(d)
Rabeprazole - - - 100μM 50μM -
HA-UBLCP1 - - + + + -
HA-DDAA - - - - - +
HA-Rpn1 - + + + + +
FLAG-Rpt1 + + + + + +
kDa
Phos-Tag
Phospho-FLAG-Rpt1
FLAG-Rpt1
37
HA-UBLCP1 /DDAA
100
HA-Rpn1
1 2 3 4 5 6
